# Association Between the Severity of Periodontal Disease and Carotid Artery Pulsatility Index in Patients with Type 2 Diabetes Mellitus

**DOI:** 10.3390/jcm15218540

**Published:** 2026-11-02

**Authors:** Sachiko Sasajima, Nobuhisa Nakamura, Megumi Miyabe, Reina Ozaki, Takeshi Kikuchi, Noritaka Sawada, Shun Kondo, Kento Kojima, Airi Hayashi, Koudai Katsumata, Koji Inagaki, Akio Mitani, Keiko Naruse

**Affiliations:** 1 Department of Internal Medicine, School of Dentistry, Aichi Gakuin University, Nagoya 464-8651, Japan; sachiko3@dpc.agu.ac.jp (S.S.); mmiyabe@dpc.agu.ac.jp (M.M.); reinaozk@dpc.agu.ac.jp (R.O.); narusek@dpc.agu.ac.jp (K.N.); 2 Division of Medical-Dental Regeneration Medicine, Center for Advanced Oral Science, Aichi Gakuin University, Nagoya 464-8650, Japan; 3 Department of Periodontology, School of Dentistry, Aichi Gakuin University, Nagoya 464-8651, Japan; tkikuchi@asahi-u.ac.jp (T.K.); nsawada@dpc.agu.ac.jp (N.S.); shunk@dpc.agu.ac.jp (S.K.); k-kojima@dpc.agu.ac.jp (K.K.); ag233d16@dpc.agu.ac.jp (A.H.); 213d09@dpc.agu.ac.jp (K.K.); kojikun@dpc.agu.ac.jp (K.I.); minita@dpc.agu.ac.jp (A.M.); 4 Department of Periodontology, Division of Oral Infections and Health Sciences, Asahi University School of Dentistry, Gifu 501-0296, Japan; 5 Department of Dental Hygiene, Junior College, Aichi Gakuin University, Nagoya 464-8650, Japan; 6 Division of Molecular Genetics and Immunology, Center for Advanced Oral Science, Aichi Gakuin University, Nagoya 464-8650, Japan

**Keywords:** diabetic complications, periodontal disease, pulsatility index

## Abstract

**Background/Objectives**: Periodontal disease is a major comorbidity of diabetes mellitus, and there is a bidirectional relationship between these two chronic inflammatory conditions. Although emerging studies have suggested that periodontal disease is associated with both macrovascular and microvascular complications of diabetes, its relationship with functional vascular parameters has not been sufficiently clarified. This study aimed to investigate the association between periodontal disease severity and vascular dysfunction, as assessed by the pulsatility index of the common carotid artery (CCA-PI), in patients with type 2 diabetes mellitus (T2DM). **Materials and Methods**: In this cross-sectional study, 60 patients with T2DM attending both diabetic complication and dental clinics were enrolled. Periodontal parameters, including probing pocket depth (PD), attachment level (AL), bleeding on probing, and periodontal inflamed surface area (PISA), were assessed. Vascular parameters were evaluated using carotid ultrasonography, with particular focus on CCA-PI. Associations between periodontal variables and vascular parameters were analyzed using Spearman’s rank correlation coefficients and multiple linear regression analyses. **Results**: The proportion of sites with PD ≥ 4 mm and AL ≥ 4 mm was positively correlated with CCA-PI. In multivariate regression analyses, only PD ≥ 4 mm remained associated with CCA-PI after adjustment for age, sex, BMI, and HbA1c. **Conclusions**: Although causality cannot be inferred from this cross-sectional study, greater periodontal disease severity, particularly the extent of periodontal pocketing, was associated with increased CCA-PI in patients with T2DM.

## 1. Introduction

Type 2 diabetes mellitus (T2DM) is a major global health problem and is associated with an increased risk of both microvascular and macrovascular complications. Periodontitis is a chronic inflammatory disease characterized by the destruction of tooth-supporting tissues. The bidirectional relationship between periodontal disease and diabetes mellitus has been extensively investigated in epidemiological and clinical studies [1,2,3]. The first large-scale study to clarify the association between type 2 diabetes mellitus (T2DM) and periodontal disease reported that the incidence of periodontitis was approximately 2.6 times higher in patients with T2DM than in non-diabetic individuals [4]. Poor glycemic control has been suggested to accelerate the progression of periodontal disease [5,6]. On the other hand, periodontal treatment has been shown to improve glycemic control in some patients with T2DM, suggesting that oral health management may contribute to comprehensive diabetes care. Consequently, increasing attention has been directed toward the systemic effects of periodontitis beyond the oral cavity.

In addition to glycemic abnormalities, the relationship between periodontal disease and diabetic complications has also been examined. In a cohort study of Pima Indians with a high prevalence of T2DM, severe periodontitis in patients with diabetes was significantly associated with higher rates of ischemic heart disease, diabetic nephropathy, and mortality than in those without periodontitis.

Our previous study suggested that periodontitis may promote the onset and progression of atherosclerosis, and that the coexistence of diabetes and periodontitis could accelerate the development of both microvascular and macrovascular complications associated with diabetes [7,8]. In contrast, nonsurgical periodontal therapy and additional systemic antibiotics in patients with peripheral arterial disease and severe periodontitis did not improve 18F-FDG PET/CT values, which can measure inflammation in the aorta, carotid, and vertebral arteries [9].

Several noninvasive vascular parameters have been proposed for assessing vascular health, including carotid intima–media thickness (IMT), pulse wave velocity (PWV), flow-mediated dilation (FMD), and carotid artery pulsatility index (CCA-PI). Among these markers, CCA-PI is considered a sensitive indicator of hemodynamic and functional vascular alterations and may detect vascular abnormalities before morphologic changes become evident. Although epidemiological studies have demonstrated associations between periodontal disease and cardiovascular disease in patients with diabetes, functional vascular parameters reflecting downstream resistance and arterial compliance, such as CCA-PI, have been less extensively investigated. Moreover, the majority of available studies have included patients with advanced periodontitis, making it unclear whether milder forms of periodontal disease are also associated with vascular dysfunction in patients with T2DM. Clarifying this relationship is clinically important because patients with diabetes often present with mild-to-moderate periodontal disease severity in routine clinical practice. Although previous studies have evaluated structural vascular markers, such as IMT and arterial stiffness indices, the relationship between periodontal disease and carotid PI remains poorly understood. Periodontitis, diabetes mellitus, and cardiovascular disease share several pathophysiological mechanisms, including chronic low-grade inflammation, oxidative stress, endothelial dysfunction, and immune dysregulation.

Therefore, clarification of the relationship between periodontal disease severity and vascular dysfunction may contribute to a better understanding of integrated medical–dental management strategies for patients with T2DM. Therefore, the present study aimed to investigate the associations between periodontal clinical parameters and vascular function, assessed using CCA-PI, in patients with T2DM. We hypothesized that greater periodontal disease severity would be associated with impaired vascular function.

## 2. Materials and Methods

### 2.1. Study Design and Population

This study included 60 patients (40 men and 20 women) who provided informed consent and had their first visit to the Department of Internal Medicine, Division of Diabetic Complications, Aichi Gakuin University Dental Hospital, between April 2015 and March 2024. During the study period, 82 patients with T2DM attending both the diabetes complications clinic and the Department of Periodontology were consecutively screened. The inclusion criteria were: (1) diagnosis of T2DM according to the criteria of the Japan Diabetes Society; (2) age ≥ 20 years; (3) availability of complete periodontal examinations and carotid ultrasonographic assessments, performed within the same clinical evaluation period; and (4) provision of written informed consent. In total, 22 patients were excluded as they were pregnant, had an active infectious disease, a current malignancy, severe systemic illness, a history of periodontal treatment within the preceding 6 months, or used antibiotics within the preceding 3 months. Individuals with incomplete clinical or periodontal data were also excluded from the analysis. Because complete periodontal assessments and carotid ultrasonographic measurements were required on the same visit, 60 patients ultimately fulfilled all eligibility criteria and were included in the final analysis. This study was conducted in accordance with the Declaration of Helsinki and complied with the Ethical Guidelines for Medical and Health Research Involving Human Subjects in Japan. The research protocol was reviewed and approved by the Human Ethics Review Committee of the School of Dentistry at Aichi Gakuin University (approval number: 404). Written informed consent was obtained from all participants prior to enrollment.

### 2.2. Data Collection

Demographic and medical information, including age, sex, body mass index (BMI), duration of diabetes, smoking status, blood pressure, and medication use, was obtained from medical records. On the day of examination, body mass index (BMI) and blood pressure were measured and recorded. Blood and urine samples were collected to measure glycated hemoglobin (HbA1c), serum creatinine (Cre), estimated glomerular filtration rate (eGFR), urinary albumin-to-creatinine ratio (UACR), and lipid profiles. Triglyceride (TG), low-density lipoprotein cholesterol (LDL-C), and high-density lipoprotein cholesterol (HDL-C) levels were measured on an automated analyzer with an AU LDL-C reagent (Beckman Coulter, Inc., Brea, CA, USA). HbA1c levels were determined by high-performance liquid chromatography using a glycohemoglobin analyzer (HLC-723G11; Tosoh Corporation, Tokyo, Japan) and expressed according to the National Glycohemoglobin Standardization Program (NGSP) criteria. Cre levels were measured using an enzymatic assay on an automated analyzer with an AU Crereagent (Beckman Coulter, Inc., Brea, CA, USA). eGFR (mL/min/1.73 m^2^) was calculated using the validated Japanese equation [10].

### 2.3. Measurement of Cardio-Ankle Vascular Index

The cardio-ankle vascular index (CAVI) was determined using a VaSera CAVI instrument (Fukuda Denshi Co., Ltd., Tokyo, Japan). Cuffs were placed on both upper arms and ankles of a subject positioned supine, with their head in a midline position. Measurements were taken after the subject had rested for 10 min. Brachial and ankle pulse waves were detected using low cuff pressures (30 to 50 mmHg) to minimize the impact of cuff pressure on hemodynamics. After measurement of the CAVI, the blood pressure measured in all four limbs to assess the ankle–brachial index (ABI).

### 2.4. Measurement of Carotid Ultrasonography

Carotid ultrasonography was used to measure the maximum and mean intima–media thicknesses (max-IMT and mean-IMT, respectively) and the pulsatility index of CCA (CCA-PI) using carotid artery echocardiography (Aprio 700; Canon Medical Systems Corp., Tochigi, Japan). The common carotid artery was evaluated bilaterally according to standardized protocols. Carotid IMT was measured at plaque-free segments of the far wall of the common carotid artery. PI was measured using duplex Doppler ultrasonography and was regarded as a surrogate marker reflecting downstream vascular resistance and arterial compliance.

Blood flow velocity waveforms were obtained with the subjects in the supine position after an adequate rest period. A Doppler sample volume was placed in the distal segment of CCA.

PI was calculated according to Gosling’s formula as follows:

$$PI\;=\;\frac{PSV-EDV}{TAMV}$$

where PSV is the peak systolic velocity; EDV is the end-diastolic velocity; and TAMV is the time-averaged mean velocity of the cardiac cycle [11]. Higher PI values indicate increased downstream vascular resistance and reduced arterial compliance, reflecting impaired vascular function.

### 2.5. Periodontal Examination

On the day of examination for diabetic complications, patients also underwent a periodontal evaluation at the Department of Periodontology, Aichi Gakuin University Dental Hospital. Periodontal examinations were performed by experienced periodontists and included the following assessments.

1. Oral hygiene status: Presence or absence of dental plaque accumulation.
2. Gingival inflammation: Presence or absence of bleeding on probing (BOP).
3. Periodontal pocket depth (PD): Distance from the gingival margin to the bottom of the pocket.
4. Attachment level (AL): Distance from the cementoenamel junction to the bottom of the pocket.
5. Periodontal inflamed surface area (PISA).

The periodontal inflamed surface area (PISA) was calculated according to the method proposed by Nesse et al. PISA was calculated using analytical software based on PD and BOP measurements obtained from periodontal examinations to quantify the surface area of the inflamed periodontal tissue. Higher PISA values indicate a greater inflammatory burden arising from periodontal tissues [12,13,14]. The diagnosis and assessment of periodontitis were conducted with reference to the 2018 Classification of Periodontal and Peri-implant Diseases and Conditions [15,16,17]. For quantitative analyses, periodontal status was characterized by using continuous clinical periodontal parameters, including PD, AL, BOP, and PISA, rather than by categorical staging and grading variables. To minimize measurement bias, periodontal examinations were performed by experienced periodontists.

### 2.6. Statistical Analysis

All statistical analyses were conducted using SPSS statistical software (version 30.0; IBM Corp., Armonk, NY, USA). The normality of continuous variables was assessed using the Shapiro–Wilk test. For regression analyses, assumptions of linearity, homoscedasticity, and normality of residuals were evaluated using residual plots and Q-Q plots. No major violations were observed. Correlations between periodontal parameters and vascular variables were evaluated using Spearman’s rank correlation coefficients because several variables were not normally distributed. To investigate independent associations between periodontal parameters and vascular indices (CAVI, ABI, and CCA-PI), multiple linear regression analyses were performed using a forced-entry method. Models were adjusted for potential confounding factors, including age, sex, BMI, and HbA1c (Model 1), with additional adjustments for diabetes duration (Model 2), for systolic blood pressure (Model 3), and for eGFR (Model 4). Continuous variables are summarized as mean ± standard deviation (SD) or median with interquartile range, as appropriate, while categorical variables are expressed as frequencies and percentages. A two-sided *p*-value < 0.05 was considered statistically significant. Owing to the limited sample size, the number of covariates included in the regression models was restricted to avoid model overfitting. Age, sex, BMI, and HbA1c were selected a priori based on their established associations with both periodontal disease and vascular dysfunction. Other cardiovascular risk factors, including smoking status, blood pressure, lipid levels, diabetes duration, and medication use, were considered potential residual confounders and thus as study limitations. The primary vascular outcome of interest was CCA-PI, which was prespecified based on the study hypothesis that periodontal disease severity may be associated with functional vascular impairment. The association between the proportion of sites with PD ≥ 4 mm and CCA-PI was specified a priori as the primary hypothesis. Other correlation analyses were considered exploratory. Because this was an exploratory study, no formal a priori sample-size calculation was performed.

## 3. Results

### 3.1. Characteristics of the Study Subjects

The characteristics of the study’s participants are summarized in Table 1. The mean age of the patients was 66.6 ± 9.4 years, comprising 40 men (66.7%) and 20 women (33.3%). The mean body mass index (BMI) was 24.1 ± 3.2 kg/m^2^, and 5.5% of the participants were current smokers. Regarding the treatment of diabetes, 15.3% of the patients were being treated with insulin and 3.4% with glucagon-like peptide-1 (GLP-1) receptor agonists, indicating that approximately 20% of the participants were receiving injectable therapies. Regarding oral medications, 13.6% were taking sodium–glucose cotransporter 2 (SGLT2) inhibitors, 35.6% angiotensin II receptor blockers (ARBs), 8.5% angiotensin-converting enzyme inhibitors (ACE-Is), 52.5% statins, and 6.8% fibrates.

Laboratory tests showed mean values for HbA1c of 7.3 ± 1.3%, Cre of 0.87 ± 0.18 mg/dL, and eGFR of 64.4 ± 12.6 mL/min/1.73 m^2^. UACR was 28.9 ± 35.2 mg/g Cr, and all participants were classified as having stage 1 or stage 2 diabetic nephropathy.

Carotid ultrasonography revealed the mean intima–media thickness (IMT), maximum (max) IMT, and CCA-PI. Although the mean IMT was below 1.1 mm, the mean max-IMT and CCA-PI exceeded 1.1 mm and 1.0, respectively.

### 3.2. Characteristics of Dental Examinations

The characteristics of the dental examinations are shown in Table 2. The mean number of present teeth was 23 ± 5.8. The average of the BOP rate was 24.4 ± 17.0 (%), the percentage of PD ≥ 4 mm was 10.2 ± 10.7%, the percentage of sites with AL ≥ 4 mm was 24.8 ± 17.5%, and PISA was 352.9 ± 292.0 mm^2^ (Table 2).

### 3.3. Correlations Between HbA1c and Periodontal Parameters

We performed Spearman’s rank correlation analyses for the duration of diabetes/glycemic control and periodontal parameters. HbA1c positively correlated with the proportion of sites with AL ≥ 4 mm (*p* = 0.014); however, there was no significant correlation between HbA1c and other periodontal parameters including those with a PD ≥ 4 mm, suggesting a positive association between glycemic control and the cumulative burden of irreversible periodontal tissue destruction.

### 3.4. Correlations Between Intima–Media Thickness, CCA-PI, and Periodontal Parameters

**Figure 1:**
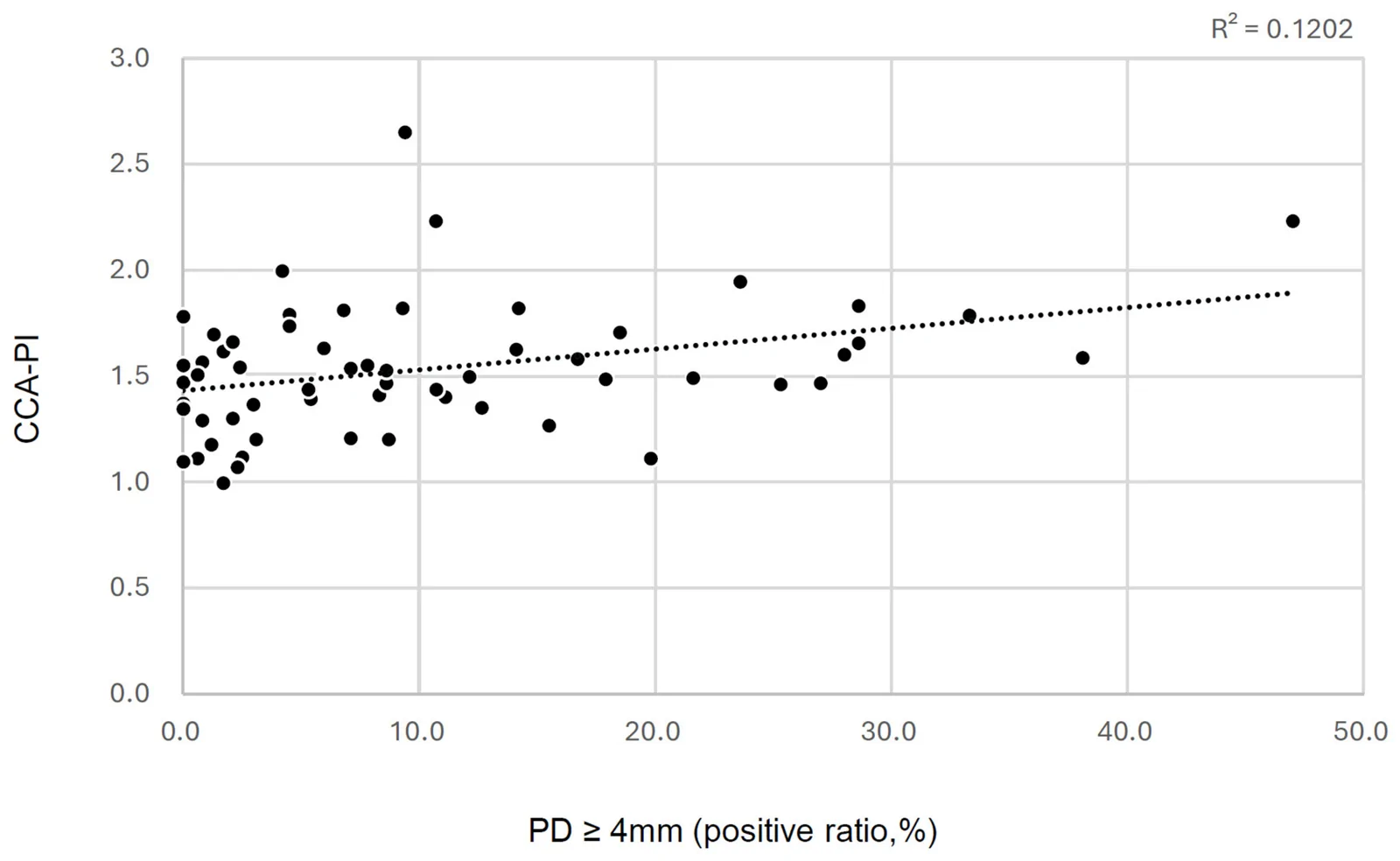
Correlation between CCA-PI and positive ratio of PD ≥ 4 mm. Scatter plot showing the relationship between the proportion of sites with PD ≥ 4 mm and CCA-PI (Spearman’s ρ = 0.36, *p* = 0.005, *n* = 60). The dashed line indicates the regression line.

**Figure 2:**
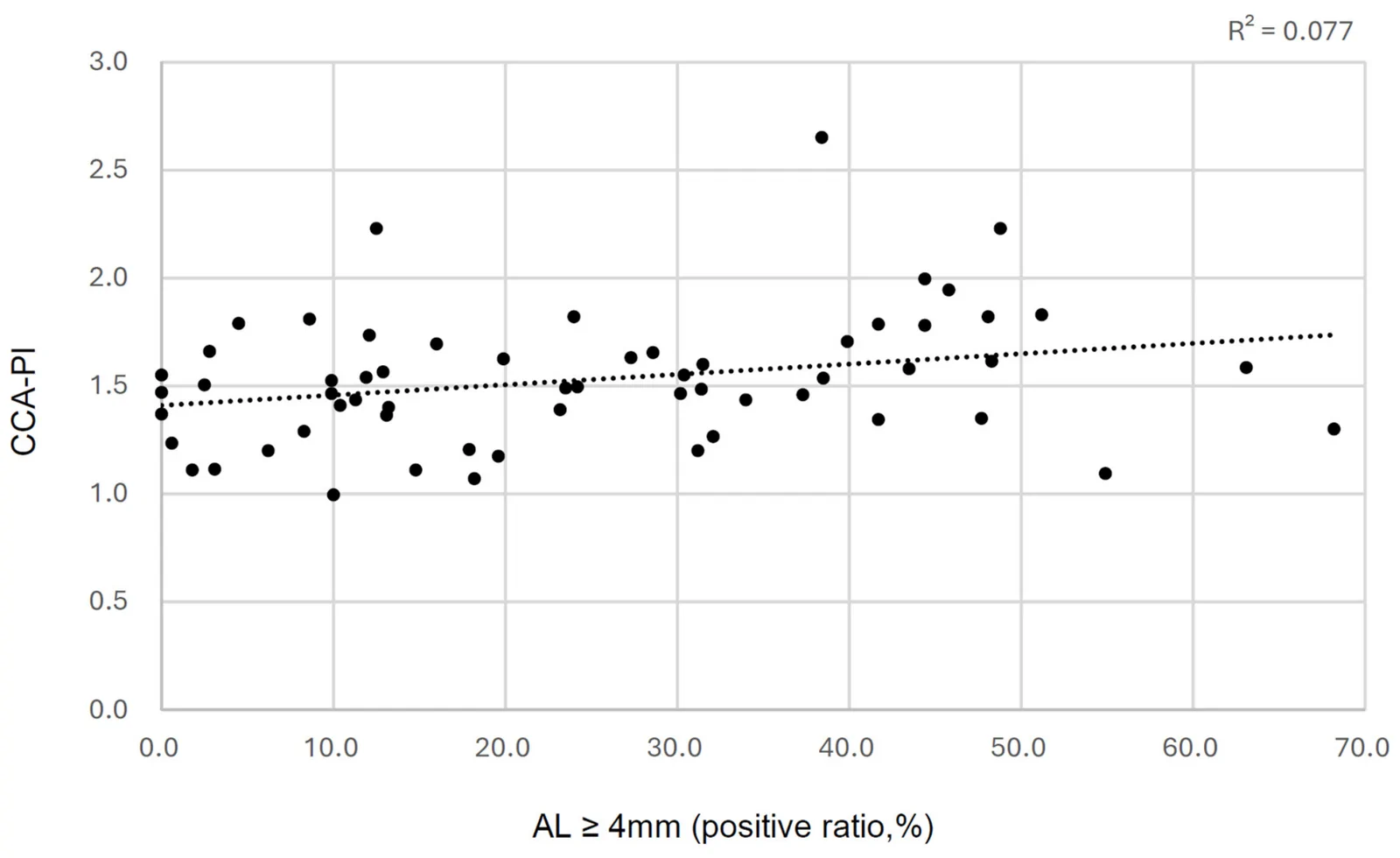
Scatter plot showing the relationship between the proportion of sites with AL ≥ 4 mm and CCA-PI (Spearman’s ρ = 0.28, *p* = 0.032, *n* = 60). The dashed line indicates the regression line.

PI reflects functional alterations in vascular resistance and arterial compliance, whereas IMT represents cumulative structural changes in the arterial wall. In our study, intima–media thicknesses (max-IMT and mean-IMT) did not correlate with periodontal parameters. On the other hand, a significant positive correlation in Spearman’s rank correlation analyses was observed between CCA-PI and PD ≥ 4 mm, as well as between CCA-PI and AL ≥ 4 mm (Figure 1 and Figure 2), suggesting that functional vascular impairment was associated with moderate periodontitis in patients with diabetes (Table 3).

### 3.5. Multiple Regression Analyses Between CCA-PI and Periodontal Parameters

The results of the multiple regression analyses adjusted for potential confounding factors, including age, sex, BMI, and HbA1c levels, are shown in Table 4. In multivariable regression analyses adjusted for age, sex, BMI, and HbA1c, the proportion of sites with PD ≥ 4 mm remained associated with CCA-PI (B = 0.008, standardized β = 0.277, 95% CI: 0.001–0.015, *p* = 0.023). The final model explained 21.1% of the variance in CCA-PI (adjusted R^2^ = 0.211). The association remained significant in the primary model and in sensitivity analyses that additionally adjusted for systolic blood pressure and eGFR, although significance was attenuated after adjustment for diabetes duration. A priori sample-size calculation was not performed because this study was exploratory in nature.

## 4. Discussion

In this exploratory cross-sectional study, we observed a positive association between the proportion of sites with PD ≥ 4 mm and CCA-PI in patients with T2DM after adjustment for age, sex, BMI, and HbA1c. However, this association was attenuated and no longer statistically significant after additional adjustment for diabetes duration. This finding suggests that diabetes duration may represent an important confounding factor and indicates that the observed association should be interpreted cautiously. Therefore, our findings should be considered hypothesis-generating rather than confirmatory.

Although CCA-PI was significantly associated with the proportion of sites with PD ≥ 4 mm, no significant associations were observed for BOP or PISA, both of which more directly reflect the current inflammatory burden of periodontal disease. Therefore, our findings may indicate a relationship between periodontal disease severity and vascular dysfunction instead of demonstrating a specific effect of periodontal inflammation itself. Chronic inflammation induced by periodontitis may promote atherosclerosis through mechanisms involving endothelial dysfunction, increased oxidative stress, and persistent elevation of inflammatory cytokines, such as IL-6, C-reactive protein (CRP), and TNF-α [18]. Periodontal disease is a chronic inflammatory condition known to induce systemic inflammation through the production of proinflammatory cytokines, such as tumor necrosis factor-α (TNF-α) and interleukin-6 (IL-6) [19]. Systemic inflammation may exacerbate insulin resistance and endothelial dysfunction in patients with diabetes [20]. Periodontal pathogens have also been detected within arterial walls [21,22], suggesting that bacteremia originating from periodontal infection may contribute to plaque destabilization through localized inflammation.

To our knowledge, this study is among the first to demonstrate an association between CCA-PI and periodontal pocket depth (PD ≥ 4 mm) in patients with type 2 diabetes. CCA-PI obtained from carotid ultrasonography reflects the difference between systolic and diastolic blood flow velocities. CCA-PI is a Doppler-derived hemodynamic parameter influenced by downstream vascular resistance, arterial compliance, cardiac function, heart rate, and systemic hemodynamic conditions, and it has been recognized as a useful marker for evaluating the risk of atherosclerosis and vascular disease [23]. Recently, elevated PI has been highlighted as a predictor of cardiovascular and cerebrovascular events [24]. In our study, patients with higher CCA-PI values exhibited a greater severity of periodontal disease, suggesting that individuals with both diabetes and periodontal disease may have an increased risk of vascular dysfunction and atherosclerosis. On the other hand, the association between PD ≥ 4 mm and CCA-PI was attenuated and no longer statistically significant after additional adjustment for diabetes duration. This finding suggests that diabetes duration may act as an important confounding factor because it is associated with both progression of periodontal disease and vascular dysfunction. Therefore, the observed relationship should be interpreted cautiously until confirmed in larger prospective studies.

Previous reports have demonstrated that patients with diabetes and periodontitis were reported to exhibit significantly greater carotid IMTs and pulse wave velocities (PWVs), which are both established markers of atherosclerosis [25,26,27]. CAVI is a noninvasive measure of arterial stiffness, and it has predictive value for cardiovascular events. On the other hand, we did not find an association between IMT and periodontal parameters.

The lack of association between periodontal parameters and carotid IMT, together with the significant association with CCA-PI, suggests that the absence of a significant association with IMT should be interpreted cautiously and does not establish that periodontal disease preferentially affects functional rather than structural vascular abnormalities. The pulsatility index reflects downstream vascular resistance and arterial compliance, which are known to be sensitive to inflammatory and endothelial dysfunction. In contrast, IMT represents cumulative atherosclerotic burden that develops over a longer time course [25,28,29]. Therefore, periodontal inflammation may contribute to early-stage vascular dysfunction before overt structural remodeling becomes apparent.

The study population generally exhibited mild-to-moderate periodontal disease severity, as reflected by the observed periodontal clinical parameters. These findings support the potential importance of periodontal assessment in patients with T2DM. However, whether periodontal treatment influences vascular, renal, or cardiovascular outcomes remains to be determined in prospective interventional studies. Importantly, the observed association between PD ≥ 4 mm and CCA-PI was identified in a population predominantly and characterized by mild-to-moderate periodontal disease severity rather than extensive advanced periodontal destruction. Despite this, significant associations with CCA-PI were observed, suggesting a possible association between mild-to-moderate periodontal disease severity and altered vascular hemodynamics in patients with T2DM. This finding has important clinical implications, as it suggests that subclinical or moderately active periodontal inflammation should not be underestimated in the management of diabetic patients [22,30,31].

Collectively, these findings indicate that the presence of periodontal disease in individuals with diabetes may be associated with an increased risk of cardiovascular events through mechanisms involving vascular dysfunction and systemic inflammation. Management of periodontal disease in patients with diabetes may be considered a component of systemic disease control [32]. On the other hand, these results underscore the importance of interdisciplinary collaboration between medical and dental professionals in the management of diabetes. Routine periodontal evaluation may provide additional information regarding vascular health; however, the clinical utility of periodontal findings as markers of vascular risk requires confirmation in larger prospective studies [33,34].

Several limitations should be acknowledged. First, because of the cross-sectional design, causality and temporal relationships cannot be determined. Second, the association between PD ≥ 4 mm and CCA-PI was attenuated and no longer statistically significant after additional adjustment for diabetes duration, suggesting potential residual confounding. Third, residual confounding cannot be excluded because several cardiovascular risk factors, including smoking history, lipid levels, and medication use, were not incorporated in the primary multivariable model, although additional sensitivity analyses were performed for diabetes duration, systolic blood pressure, and eGFR. Fourth, inflammatory biomarkers, including CRP, IL-6, and TNF-α, were not measured; therefore, the proposed inflammatory mechanism remains speculative. Fifth, the long recruitment period may have introduced temporal heterogeneity. Finally, multiple comparisons were performed, increasing the possibility of type I errors. Furthermore, the attenuation of the association after adjustment for diabetes duration suggests that the observed relationship may be partly explained by shared disease chronicity rather than an independent effect of periodontal disease. Therefore, the present findings should be regarded as hypothesis-generating rather than evidence supporting a causal pathway linking periodontal disease severity with vascular dysfunction. Confirmation in larger longitudinal and interventional studies is warranted.

## 5. Conclusions

In this exploratory cross-sectional study, a higher proportion of sites with PD ≥ 4 mm was associated with higher CCA-PI in the primary adjusted model. However, the association was attenuated and no longer statistically significant after additional adjustment for diabetes duration, suggesting potential confounding by disease chronicity. Therefore, these findings should be interpreted cautiously and regarded as hypothesis-generating. Larger prospective and interventional studies are needed to determine whether periodontal disease contributes independently to vascular dysfunction in patients with T2DM.

## Figures and Tables

**Table 1:** Characteristics of the patients.

	Value (*n* = 60)
Age (years)	66.6 ± 9.3
Sex (male/female)	40/20
BMI	24.1 ± 3.2
Current smoker (%)	5.5
Duration of diabetes (years)	11.7 ± 10.1
Insulin users (%)	15.3
GLP-1 users (%)	3.4
OHA users (%)	
SU	18.6
Glinide	5.1
DPP-4 inhibitor	61.0
Biguanide	44.1
SGLT2 inhibitor	13.6
Antihypertensive drug users (%)	
Ca blocker	33.9
ARB	35.6
ACE inhibitor	8.5
Lipid-lowering drug users (%)	
Statin	52.5
Fibrate	6.8
HbA1c (%)	7.3 ± 1.3
Cre (mg/dL)	0.87 ± 0.2
eGFR (mL/min/1.73 m^2^)	64.4 ± 12.6
Urine albumin/creatinine ratio (mg/gCr)	29.0 ± 35.2
HDL-C (mg/dL)	58.2 ± 16.7
LDL-C (mg/dL)	115.4 ± 30.8
TG non-fasting (mg/dL)	177.1 ± 106.9
CAVI	8.74 ± 1.23
ABI	1.13 ± 0.09
Max-IMT (mm)	1.37 ± 0.42
Mean-IMT (mm)	0.95 ± 0.21
CCA-PI	1.53 ± 0.31

Data are presented as Mean ± SD. Body mass index, BMI; glucagon-like peptide-1, GLP-1; oral hypoglycemic agent, OHA; sulfonylurea, SU; dipeptidyl peptidase-4, DPP-4; sodium–glucose co-transporter 2, SGLT2; calcium channel blocker, Ca Blocker; angiotensin II receptor blocker, ARB; angiotensin-converting enzyme, ACE; creatinine, Cre; estimated glomerular filtration rate, eGFR; maximum and mean intima–media thicknesses, max IMT and mean IMT; cardio-ankle vascular index, CAVI; ankle–brachial index, ABI; common carotid artery pulsatility index, CCA-PI.

**Table 2:** Results of dental examinations.

	Value (*n* = 60)
Number of present teeth	23.1 ± 5.8
BOP (positive ratio, %)	24.4 ± 17.0
PD ≥ 4 mm (positive ratio, %)	10.2 ± 10.7
AL ≥ 4 mm (positive ratio, %)	24.8 ± 17.5
PISA (mm^2^)	352.9 ± 292.0

Data are presented as Mean ± SD. Bleeding on probing, BOP; probing pocket depth, PD; attachment level, AL; periodontal inflamed surface area, PISA.

**Table 3:** Correlations between periodontal and vascular parameters.

	Number of Present Teeth	BOP (Positive Ratio, %)	PD ≥ 4 mm (Positive Ratio, %)	AL ≥ 4 mm (Positive Ratio, %)	PISA (mm^2^)
	*p*	*p*	*p*	*p*	*p*
CAVI	0.397	0.140	0.374	0.856	0.184
ABI	0.485	0.120	0.056	0.434	0.185
Max-IMT	0.814	0.808	0.722	0.052	0.769
Mean-IMT	0.325	0.784	0.927	0.217	0.925
CCA-PI	0.996	0.959	0.005 **	0.032 *	0.751

Analysis method: Spearman’s rank correlation analyses. * *p* < 0.05, ** *p* < 0.01. Bleeding on probing, BOP; probing pocket depth, PD; attachment level, AL; periodontal inflamed surface area, PISA; cardio-ankle vascular index, CAVI; ankle–brachial index, ABI; maximum and mean intima–media thicknesses, max-IMT and mean-IMT; pulsatility index of common carotid artery, CCA-PI.

**Table 4:** Multiple regression analyses of the association between CCA-PI and periodontal parameters. (**a**) Model 1 (primary model) adjusted for age, sex, BMI, and HbA1c. (**b**) Model 2 (sensitivity analysis; additional adjustment for diabetes duration). (**c**) Model 3 (sensitivity analysis; additional adjustment for systolic blood pressure). (**d**) Model 4 (sensitivity analysis; additional adjustment for eGFR).

Variable	B	SE	Standardized β	95% CI	*p*-Value
(**a**)					
PD ≥ 4 mm sites (%)	0.008	0.003	0.277	0.001 to 0.015	* 0.023
Age (years)	0.006	0.004	0.180	−0.002 to 0.014	0.157
Sex (male = 1)	−0.223	0.076	−0.348	−0.374 to −0.071	** 0.005
BMI (kg/m^2^)	0.004	0.012	0.037	−0.021 to 0.028	0.769
HbA1c (%)	−0.006	0.011	−0.061	−0.029 to 0.017	0.605
(**b**)					
PD ≥ 4 mm sites (%)	0.005	0.004	0.184	−0.002 to 0.012	0.150
Diabetes duration (years)	0.008	0.004	0.248	0.000 to 0.016	0.064
(**c**)					
PD ≥ 4 mm sites (%)	0.008	0.003	0.270	0.001 to 0.014	* 0.028
Systolic blood pressure (mmHg)	0.001	0.002	0.074	−0.003 to 0.006	0.544
(**d**)					
PD ≥ 4 mm sites (%)	0.008	0.003	0.277	0.001 to 0.015	* 0.024
eGFR (mL/min/1.73 m^2^)	−0.001	0.003	−0.027	−0.007 to 0.006	0.835

R^2^ = 0.278, adjusted R^2^ = 0.211. R^2^ = 0.365, adjusted R^2^ = 0.288. R^2^ = 0.283, adjusted R^2^ = 0.202. R^2^ = 0.279, adjusted R^2^ = 0.197. Analysis method: Multiple regression analysis. * *p* < 0.05, ** *p* < 0.01. Model 1 = primary model, Model 2 = additional adjustment for diabetes duration, Model 3 = additional adjustment for SBP, Model 4 = additional adjustment for eGFR.

## Data Availability

The data that supports the findings of this study are available from the corresponding authors upon reasonable request.

## Abbreviations

The following abbreviations are used in this manuscript:

T2DM	Type 2 Diabetes Mellitus
CCA-PI	Common Carotid Artery Pulsatility Index
CCA	Common Carotid Artery
PI	Pulsatility Index
PD	Probing Pocket Depth
AL	Attachment Level
BOP	Bleeding on Probing
PISA	Periodontal Inflamed Surface Area
BMI	Body Mass Index
HbA1c	Hemoglobin A1c
Cre	Creatinine
eGFR	Estimated Glomerular Filtration Rate
UACR	Urinary Albumin-to-Creatinine Ratio
TG	Triglyceride
LDL-C	Low-Density Lipoprotein Cholesterol
HDL-C	High-Density Lipoprotein Cholesterol
CAVI	Cardio-Ankle Vascular Index
ABI	Ankle–Brachial Index
IMT	Intima–Media Thickness
max-IMT	Maximum Intima–Media Thickness
mean-IMT	Mean Intima–Media Thickness
PSV	Peak Systolic Velocity
EDV	End-Diastolic Velocity
TAMV	Time-Averaged Mean Velocity
GLP-1	Glucagon-Like Peptide-1
OHA	Oral Hypoglycemic Agent
SU	Sulfonylurea
DPP-4	Dipeptidyl Peptidase-4
SGLT2	Sodium–Glucose Cotransporter 2
ARB	Angiotensin II Receptor Blocker
ACE (ACE-I)	Angiotensin-Converting Enzyme (Inhibitor)
PWV	Pulse Wave Velocity
TNF-α	Tumor Necrosis Factor-α
IL-6	Interleukin-6
CRP	C-Reactive Protein
PET/CT	Positron Emission Tomography/Computed Tomography
NIDDM	Non-Insulin-Dependent Diabetes Mellitus
